# Booting up the organism during development: Pre-behavioral functions of the vertebrate brain in guiding body morphogenesis

**DOI:** 10.1080/19420889.2018.1433440

**Published:** 2018-02-15

**Authors:** Celia Herrera-Rincon, Michael Levin

**Affiliations:** Allen Discovery Center, and Department of Biology, Tufts University, Medford, MA, USA

**Keywords:** brain, birth defects, embryogenesis, patterning, teratogen

## Abstract

A recent study in *Xenopus laevis* embryos showed that the very early brain has important functions long before behavior. While the nascent brain is being constructed, it is required for normal patterning of the muscle and peripheral nerve networks, including those far away from the head. In addition to providing important developmental signals to remote tissues in normal embryogenesis, its presence is also able to render harmless exposure to specific chemicals that normally act as teratogens. These activities of the early brain can be partially compensated for in a brainless embryo by experimental modulation of neurotransmitter and ion channel signaling. Here, we discuss the major findings of this paper in the broader context of developmental physiology, neuroscience, and biomedicine. This novel function of the embryonic brain has significant implications, especially for understanding developmental toxicology and teratogenesis in the context of pharmaceutical and environmental reagents.

## Introduction

A computer, automobile, or any other man-made object is generally expected to function only *after* it rolls off the assembly line – the system is first booted up and functions after its construction is complete. But what about the self-assembling process of embryogenesis – when do organs first operate during development? A recent paper [1] shows that the brain does not wait for its construction to be complete: rather, it is a remarkable early example of a complex structure that is functional during the very earliest stages of its self-assembly *de novo*.

It is widely taught in embryology texts that the heart is the first functional organ to form. However, it is now seen that the brain has functions long before behavior and appears to be providing important signals for developmental patterning as early as two days after fertilization in the *Xenopus laevis* frog embryo.

**Figure 1.**
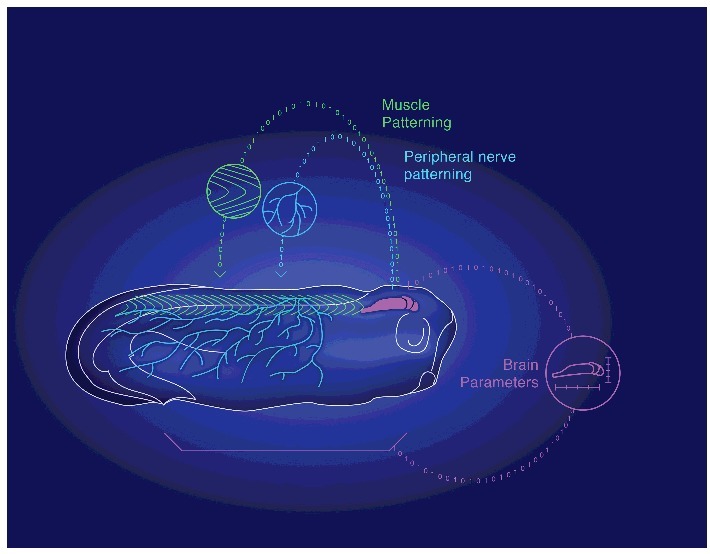
The processes of embryogenesis instructing patterning form a closed-loop control system between the brain and the body. A schematic drawing of a developing *Xenopus* embryo, representing how the embryonic brain (purple) is receiving instructive inputs from different (distal and proximal) body tissues to help building the right brain parameters (for example, shape and size). We have recently shown that this control operating system is bi-directional. The very early brain itself has, in turn, an instructive role on morphogenesis and patterning of remote tissues, such as peripheral neural network (blue) and somitic myotome (green). This closed-loop between brain and body is the earliest example of scaling and effective communication for self-assembly of complex biological structures.

It was already known that the frog embryo's brain receives input from many body tissues, including distant cells in the gut, which help the brain to form with the right shape and size [2,3]. Thus, scaling and morphogenesis of the brain are themselves instructed by other tissues. But, it turns out that this set of control mechanisms is bi-directional: the brain itself is regulating patterning of remote body tissues, in a control loop that operates at the very earliest stages of development (Figure 1).

### When does the brain start working? Long before we thought.

**Figure 2.**
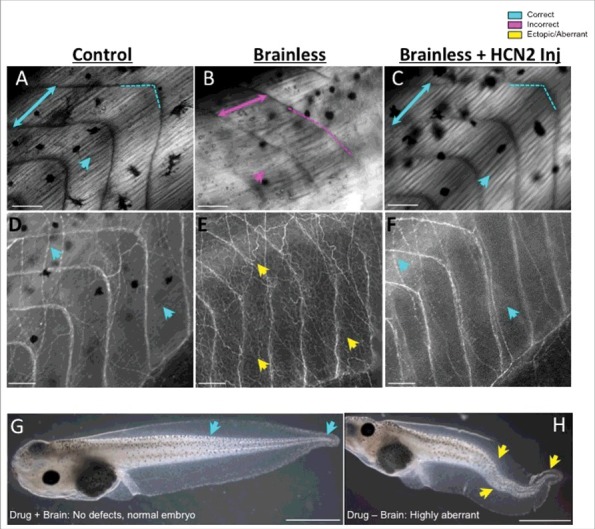
Functions of the very early brain include guiding and protecting correct embryo morphogenesis, which can be mimicked by ectopic expression of ion channels. Comparative images of muscle (A-C, under polarized light) and nerve (D-F, revealed by immunofluorescence against acetylated-alpha tubulin) structures of embryos that developed with a brain (Control), embryos that developed without a brain (Brainless), and embryos that developed without a brain and were injected with messenger RNA encoding the HCN2 ion channel (Brainless + HCN2 Inj). The absence of a brain during development leads to abnormal development of the muscles and the peripheral nerves, at considerable distances from the head, with disorganization of the body plan, myotomes lacking proper angles (magenta-dashed line in B), alterations in somite length (represented by two-headed arrows) and ectopic growth of nerve tissue (yellow arrows in E). By manipulating bioelectricity, for example via the mis-expression of HCN2, rescues the devastating muscle and nerve mispatterning that occurs in brainless animals (turquoise arrows in C, F). Rostral is right and dorsal is up. Scale bar = 100 μm. G, H. The effects of the drug (*RS*)-(tetrazol-5-yl)glycine during development depends on the presence or absence of the nascent brain in the embryo. While exposure to this drug does not cause developmental defects in control embryos (turquoise arrows in G indicate correct tail phenotype), it leads to severe deformities (yellow arrows in H demarcate bent spinal cord and tail aberrations) when the brain is not present to protect. Rostral is left and dorsal is up. Scale bar = 1 mm.

The recent findings, which make use of a simple surgical brain amputation process (followed by extensive molecular and cellular analysis) can be summarized as follows (Figure 2).

(1) Absence of the early brain leads to muscle and peripheral nerve mispatterning (defects). Animals that developed without a brain exhibited abnormal patterns of segmented embryonic tissues known as somites, and aberrations in organization and structure of the trunk muscle fibers (Figure 2A–2C; lower collagen density; shorter/longer myotomes with greater angles, leading to the lack of the typical chevron-shape; and overall displacement of the body axis, with highly bent tails). Development of the peripheral nervous system was also profoundly altered in the brainless embryos, with intense and disorganized ectopic growth of internal nerve fibers in the entire animal body (Figure 2D–2F). The aberrant nerve sprouting was not due to deficiencies of a putative pruning phase, as we showed that it occurs long before the endogenous pruning of the peripheral innervation in *Xenopus* embryos. One interesting observation is that the nascent brain affects peripheral nerve formation via the spinal cord, but uses a different pathway – one that does not involve the spinal cord – to influence muscle patterning.
(2) Absence of the early brain causes embryos to be much more sensitive to certain drugs, turning otherwise harmless compounds into potent teratogens. For example, while the NMDA glutamate receptor agonist (*RS*)-(tetrazol-5-yl)glycine [4] had no effect on normal embryos, it provoked severe deformities in animals developing without a brain (such as highly bent notochords and multiple ‘pigtail’ spiraling at the tip of the tail; Figure 2G & 2H). This experiment indicated that a normal brain serves to protect developmental patterning from drug-induced effects that otherwise result in serious abnormalities.
(3) The functions of the early brain can be mimicked by artificially providing signals via neurotransmitters and bioelectric activity of ion channel proteins. Specifically, the phenotypes induced by early absence of brain can be partially rescued in brainless animals by neurotransmitter drugs and by misexpression of Hyperpolarization-activated Cyclic Nucleotide-gated 2 (HCN2) ion channels (Figure 2C & 2F). Scopolamine treatment (an anticholinergic agent that inhibits the activity of the muscarinic acetylcholine receptor [5] immediately after brain removal and applied continuously during the first week, prevented the defects in segmented muscle tissues – both in terms of organization and size. Specific alterations in the bioelectric state, by ectopic expression of the HCN2 ion channel [6] also counteracted the effects of a missing brain on somitic myogenesis and neural development, allowing fully organized muscle fibers and peripheral neural network. The HCN2-rescue effects acted on tissues that were not themselves expressing the exogenous HCN2 (a non-cell-autonomous effect), as injecting the mRNA encoding the channel in only one half of the embryo was sufficient to protect the contralateral side. Further analysis of HCN2 expression within neural and non-neural cells suggested that the modulation of bioelectrical states within the neural structures mimics endogenous signaling from the brain during morphogenesis and patterning.

### Probing long-range patterning control loops

Establishment of this brain amputation assay exploited unique advantages of the *Xenopus* embryo model system. For example, mouse mutants with genetically-induced brain defects would not have been ideal for this work because many brain-specific genes are widely expressed during embryogenesis, and any mutation could have directly affected numerous tissues. By surgically removing the brain in a genetically wild-type background, it was possible to cleanly study the effects of the brain itself on normal tissues. The optical, surgical, and pharmacological accessibility of the frog embryo, and the ability to remove the brain at a precisely-defined time-point in development is a uniquely advantageous aspect of the *Xenopus* model. At the same time, these data are likely to be relevant beyond frog embryos, as the *Xenopus* model system has many conserved molecular and anatomical features with mammalian models and is used for biomedically-relevant research in areas of birth defects, [7–9] immunology [10], neuroscience [11,12], regenerative medicine [13,14], and cancer [15].

Importantly, the observed defects occurred in areas very far from the brain, suggesting that the effect is not simply due to local mechanical damage that can affect morphogenesis in that region. This instead suggests the existence of specific long-range signaling mechanisms by which developmental information is delivered to remote areas by organizing structures such as brains and begs the question: might there be others remaining to be discovered? Such long-range mechanisms, even at early stages before an extensive circulation and hormonal system is available to spread chemical messages body-wide, have already been observed – for example, in the field of cancer, where decisions as to whether an oncogene-bearing group of cells will make a tumor or not is a function of the physiological state of remote tissues [16,17].

Innervation has traditionally considered to be formed long before it becomes functional, remaining in a *quiescent* state until a stimulus triggers response [18]. Could the early embryonic brain be acting like a relevance indicator to the rest of the cells/tissues/systems in the body? The nascent brain might be an ‘organizer’, communicating to the rest of the body (secondary systems like muscle and nerves) by long-distance signals when a significant developmental event has occurred, closing the control loop for proper morphogenesis. This might explain why a relatively low information-content treatment, like neurotransmitter drug soaking, can mimic the protective effects of the brain on morphogenesis. For example, Strauss *et al*. [19] activated the silent (but fully formed) putative neural substrate implicated in lung breathing in *Xenopus* larvae just by using a GABA receptor antagonist (developmental disinhibition). One straightforward consequence of this hypothesis is the possibility of finding the signals/events that trigger the activation (or disinhibition) of one specific brain signaling, and, in turn, pathways and molecules by which the brain responds. Understanding this could be relevant to therapeutic applications (specific treatments in potential hazardous situations that mimic and/or push one specific brain response) and in morphogenetic engineering.

### Open questions

Many interesting questions for future work are raised by these studies. First, what other organs/structures require the presence of the brain? Extensive analysis of other tissue systems and markers is likely to reveal additional targets beyond the PNS and muscle. Conversely, what other organs besides the brain might provide instructive influences? The development of specific ablation strategies – chemical and optical [20,21], in addition to microsurgical – will facilitate the search for additional organizing centers. With respect to the brain-derived signaling, it will be important to not only better characterize the molecular mechanisms by which it instructs remote regions, but even more importantly to gain an understanding of the meaning (information content and degree of pattern encoding) of the signals. This will directly enable biomedical mimicry of such signals to improve outcomes in birth defects, injury response, and *in vitro* bioengineering of synthetic morphology. The extensive use of ion channel and neurotransmitter hardware to implement control of complex structure and function is a clear pointer to the common evolutionary origin of somatic and neural cognition [22,23]. In this sense, these experiments are part of an emerging field in which the spatial (patterning) and temporal (behavioral) information is integrated, blurring the boundaries between brain and body [24–27].

### Implications for therapeutics

These data have many potential implications for biomedicine. The fact that the affected areas are far away from the normal site of the brain suggests long-range interactions that could be capitalized upon, to provide surrogate-site diagnostics or interventions for hard-to-reach anatomical locations that could be applied elsewhere – especially relevant in neurotherapeutic applications. This also suggests that when looking for etiologies of specific birth defects, it is important to not restrict the suspected area to the locality of the damage site – the culprit could be in another part of the body. This provides a daunting view of the complexity of understanding and repairing defects. However, there is a silver lining here, even beyond the possibility of more convenient treatment or diagnostic locales. Might it be possible to provide brain-like signals to augment patterning and healing responses, by co-culturing with a mini-brain or brain organoid? For example, to help integration of transplants, improve patterning of regeneration therapies, shield embryos from teratogenic insults, or improve the patterning of synthetic biological structures *in vitro*? While (temporary) implants of brain tissue or brain organoids is certainly in the realm of possible therapies even *in vivo*, that may not be necessary. The original study showed that the brainless phenotype could be ameliorated by a rather limited number of brain-like influences (bioelectric and neurotransmitter interventions); it may not require an entire brain to recapitulate the important signaling dynamics. Thus, the potential therapeutic implications of this work extend beyond the embryo and toward regenerative medicine and synthetic biology of large-scale structures [28–30].

### Birth defects: Context is crucial

A key area of impact for these new findings is likely in teratology and the etiology of birth defects. One of the biggest questions in this field is why many compounds induce defects in some embryos but not others. In some cases, the percentage of affected individuals is so low that it confounds the issue of whether the reagent involved is a teratogen; for example, in the case of serotonin reuptake inhibitors, which affect a set of serotonergic pathways that are important for neural and non-neural embryogenesis [31–33], but are not yet officially labeled as teratogens [34–37]. The finding that the compound (*RS*)-(tetrazol-5-yl)glycine has no effect on wild-type embryos but causes drastic defects in brain-deficient embryos suggests a more nuanced understanding of teratogenesis. A compound may be safe for many embryos, but may dramatically affect others whose brain patterning signals are delayed or impaired by genetics or other physiological/environmental factors. Thus, it may be simplistic to assign categories of ‘teratogenic’ or ‘safe’ to a specific compound itself: whether or not it will disrupt embryonic development may be a function of the embryo's state as much as the drug itself.

While it is widely acknowledged that susceptibility varies within populations, this knowledge is not generally used in any practical way to guide fetal exposure scenarios or formulate regulatory practices. The novel brain patterning data show that impairment of brain function can lower the threshold for otherwise innocuous drugs to cause severe defects; this suggests that the developmental toxicology of the future may be able to predict the conditions under which specific chemicals pose significant dangers; it will indicate not merely whether a compound is ‘safe’ during pregnancy, but also specify how much risk it poses given specific environmental and genetic co-factors. Better yet, it may be possible to use specific neurotransmitter or ion channel-modulating drugs as anti-teratogens: augmenting brain signaling to provide a degree of protective influence in high-risk cases where fetal exposure is medically necessary. We are currently screening such interventions for the ability to ameliorate a wide range of common teratogens.

Another interesting field in which our findings could have an impact is in early-life stress (both prenatal and postnatal). Prenatal and postnatal stress has been traditionally related to neurophysiological, neuroendocrine and cognitive alterations (neurotransmitter levels [38], dysregulation of the hypothalamus-pituitary-adrenal axis [39], and intimate relationship to the psychopathology [40–42] in postnatal life (reviewed in References [43,44]). Prenatal stress is also associated in the adult offspring with cardiovascular [45], metabolic [46], immune, [47] and reproductive [48] dysfunctions. But what about the potential implications of early stress on patterning/morphogenesis of the embryo? Recently, cognitive defects in the offspring due to maternal stress have been shown to be related to an impaired neurogenesis during development in mammals [41]. This relationship seems to be well-conserved, as a recent paper using young zebrafish showed that stress exposure has an impact on the brain size, leading to smaller brain volume [49] (similar results have been found after prenatal ethanol exposure [50,51]). However, no prior studies have focused on patterning defects at distant regions from the brain. Considering our results, early stress, or any other condition affecting neurotransmitters and brain signaling in the embryo, could have a direct impact on long-range morphogenesis. The nascent brain should not be necessarily considered as the final target of any neuroactive drug or condition, since it is sending out signals to guide large-scale patterning and formation and the consequences may spread widely throughout the many tissue patterns of the developing body.

### Future prospects

The finding that the brain instructs morphogenesis during its self-assembly has many implications for regenerative medicine and developmental biology. Moreover, it reveals perhaps the earliest known example of the brain-body interface. This connects the developmental biology context to the emerging field of primitive cognition [52–58] and neuroplasticity [59–62]. Understanding how structures process information and instruct other events while their own hardware is being remodeled will impact our fundamental understanding of how memories are encoded in biological media, and thus how brains might keep memories during the inevitable development of therapies for massive brain remodeling and regeneration [63]. Ultimately, it may also be possible to exploit the tightly-integrated feedback loop between brain function and body morphology for engineering robust self-repairing bio- or artificial robotics, via the development of unconventional computing platforms based on biological principles [64–69]. Thus, analysis of model systems that facilitate analysis of the events at the origin of both mind and body [70] is likely to enrich both basic biology and biomedicine in a wide range of fundamental directions.
